# Circulating miR-122-5p, miR-125b-5p, and miR-27a-3p in Post-Mortem Whole Blood: An Exploratory Study of the Association with Sepsis-Related Death

**DOI:** 10.3390/cimb48010049

**Published:** 2025-12-30

**Authors:** Carla Occhipinti, Andrea Scatena, Emanuela Turillazzi, Diana Bonuccelli, Paolo Pricoco, Marco Fornili, Aniello Maiese, Stefano Taddei, Marco Di Paolo, Anna Rocchi

**Affiliations:** 1 Department of Surgical Pathology, Medical, Molecular and Critical Area, Institute of Legal Medicine, University of Pisa, 56126 Pisa, Italy; 2 Forensic Medicine Unit, North-West Tuscany Local Health Autority, 56121 Lucca, Italy; 3 Department of Clinical and Experimental Medicine, Unit of Medical Statistics, University of Pisa, 56126 Pisa, Italy; 4 Department of Anatomical, Histological, Forensic and Orthopedic Sciences, Sapienza University of Rome, Viale Regina Elena 336, 00161 Rome, Italy; 5 Department of Clinical and Experimental Medicine, University of Pisa, 56126 Pisa, Italy

**Keywords:** circulating miRNAs, post-mortem sepsis diagnosis, forensic molecular pathology

## Abstract

Accurate post-mortem diagnosis of sepsis remains a critical challenge in forensic pathology, as conventional morphological findings often lack specificity. Circulating microRNAs (miRNAs) have been proposed as stable molecular biomarkers, yet their diagnostic value in cadaveric samples is still unclear. This exploratory study investigated the expression of three candidate miRNAs (miR-122-5p, miR-125b-5p, and miR-27a-3p) in post-mortem peripheral whole blood to assess their association with sepsis-related death versus non-infective controls. Out of 58 cases, 45 met quality-control criteria (26 sepsis-related deaths and 19 controls). miRNA expression was quantified by qRT-PCR, normalized to miR-320, and analyzed using ΔCt values. Group differences were evaluated using linear regression models with adjustment for age, sex, and post-mortem interval, with Benjamini–Hochberg correction for multiple testing. In adjusted models, miR-125b-5p and miR-27a-3p showed evidence of association with sepsis status, whereas miR-122-5p did not. These results support the feasibility of miRNA quantification in post-mortem samples and motivate validation in larger, independent cohorts and within multimodal post-mortem diagnostic frameworks.

## 1. Introduction

According to the World Health Organization (WHO), sepsis is a syndrome characterized by an excessive inflammatory response to infection, most often triggered by bacteria, but also caused by viruses and, less commonly, by parasites or fungi. This overwhelming response can lead to tissue and organ damage in the host, potentially resulting in shock, multiple organ failure, and death.

To date, sepsis affects nearly 49 million people worldwide each year, with 11 million associated deaths, accounting for approximately 20% of global mortality [1]. These data highlight the enormous global burden of sepsis, which continues to pose a major challenge for healthcare systems worldwide.

Despite its clinical relevance, an accurate diagnosis of sepsis remains challenging, both in the clinical setting, where early recognition is crucial for effective treatment (but is often hindered by its nonspecific and overlapping manifestations), and in forensic pathology, where the absence of specific post-mortem markers makes its identification even more difficult [2]. Autopsies therefore represent a key tool in cases of suspected sepsis-related death, both to establish the cause of death and to evaluate whether issues in patient management or potential medical malpractice may have contributed [3].

However, the lack of specific morphological markers has driven increasing interest in the search for novel diagnostic approaches. Among these, molecular biomarkers such as microRNAs (miRNAs) have emerged as promising candidates, given their stability in biological samples and their involvement in the regulation of inflammatory and immune responses.

MiRNAs are small, single-stranded, non-coding RNA molecules, typically 21–23 nucleotides in length, that regulate post-transcriptional gene expression. They are ubiquitous, as they play a key role in numerous biological processes, such as cell differentiation, proliferation, apoptosis, and immune response [4].

Recent studies have shown that distinct miRNA expression patterns are associated with sepsis, reflecting both the host inflammatory response and organ dysfunction, thereby suggesting their potential role as diagnostic and prognostic biomarkers. However, the vast majority of this evidence derives from studies conducted on living patients, whereas their investigation in post-mortem settings appears to be limited to a few reports [5,6].

Despite their potential, several limitations must be considered before miRNAs can be reliably implemented as biomarkers of sepsis. A major issue is the variability in expression profiles reported across studies, which may reflect differences in patient populations, sample types, collection and storage conditions, or analytical techniques. Furthermore, although numerous dysregulated miRNAs have been described in sepsis, their precise mechanistic roles and downstream targets remain incompletely understood [7]. In forensic applications, additional challenges arise: post-mortem changes, RNA degradation, and tissue-specific variability may compromise the detection of certain miRNAs, thereby limiting their diagnostic utility after death [8,9].

Given these considerations, and the limited exploration of miRNA expression in post-mortem samples, further investigation is warranted to determine which miRNAs may be informative in the forensic context and how they could contribute to improving the diagnosis of sepsis. In light of this, the present study investigates the post-mortem expression of three selected circulating miRNAs (miR-122-5p, miR-125b-5p, and miR-27a-3p) in cases of sepsis-related death, in order to assess their potential association with sepsis and their possible utility as biomarkers in the forensic setting.

The three miRNAs investigated (miR-122-5p, miR-125b-5p, and miR-27a-3p) were selected based on prior evidence in the clinical literature showing their involvement in sepsis and their association with poor outcome. All three have been reported to display higher circulating levels in patients with a fatal course (non-survivors) compared with survivors across independent cohorts, suggesting potential relevance as severity- and prognosis-related biomarkers—an aspect particularly pertinent in a post-mortem forensic setting [10,11,12,13]. From a mechanistic standpoint, these candidates also map onto complementary processes implicated in lethal sepsis: miR-125b-5p has been linked to dysregulated innate immune and inflammatory signaling (including NF-κB-related pathways) [14,15,16], miR-122-5p to organ injury and systemic derangements (notably hepatic dysfunction with downstream coagulation/inflammatory imbalance) [17,18], and miR-27a-3p to endothelial activation and immune–oxidative stress modulation [13,19,20]. Taken together, the association with mortality and the convergence with sepsis-relevant biology motivated their inclusion in the present study.

## 2. Materials and Methods

### 2.1. Case Selection and Sample Collection

A total of 58 peripheral whole-blood samples were obtained from cases undergoing medico-legal autopsy ordered by the Public Prosecutor as part of routine forensic proceedings; the samples were collected for medico-legal purposes and retrospectively analyzed for this study. No additional procedures were performed for research purposes. Whole blood (1–2 mL) was collected by puncture with needle and syringe, preferably into EDTA tubes, and immediately stored frozen until assayed. Whole blood was chosen because it can be reliably obtained in routine forensic autopsies, even when plasma/serum separation is not feasible (e.g., due to hemolysis or limited sample volume), and because it provides a systemic matrix that may capture both extracellular and cell-associated miRNA fractions reflecting processes occurring before death; in contrast, organ tissues show marked organ-specific expression patterns and would require different sampling and normalization strategies.

The study population included 32 individuals (15 women and 17 men) who died with an antemortem diagnosis of sepsis. The antemortem diagnosis was retrieved from hospital medical records and was established by the treating physicians according to the Surviving Sepsis Campaign recommendations in use at the time [21], based on evidence of suspected/confirmed infection together with systemic involvement and organ dysfunction (including clinical course, laboratory findings, microbiological cultures and/or imaging when available, and treatment for sepsis).

The control group consisted of 26 adults (4 women and 22 men) who died of non-infective causes, without antemortem clinical suspicion of infection and without macroscopic or histological evidence of infection at autopsy. Deaths in the control group were characterized by a sudden mode of onset.

### 2.2. RNA Isolation and miRNA Analysis

Total RNA was extracted using MagMAX™ *mir*Vana™ Total RNA Isolation Kit (Applied Biosystems, Carlsbad, CA, USA) following the instructions of the manufacturer at the section “*Isolate RNA from Whole blood samples*”. The purity and quantity of RNA were assessed with NanoDrop™ 2000 Spectrophotometers (ThermoFisher Scientific, Carlsbad, CA, USA). The samples were used immediately or stored at −80 °C until assayed.

Complementary DNA (cDNA) was synthesized using the TaqMan^®^ Advanced miRNA cDNA Synthesis Kit (Applied Biosystems, Carlsbad, CA, USA), according to manufacturer’s manual. Quantitative real-time PCR (qRT-PCR) was carried out using TaqMan Fast Advanced Master Mix (Applied Biosystems, Carlsbad, CA, USA) and the detection of each miRNA was performed using TaqMan Advanced miRNA Assays (Applied Biosystems, Carlsbad, CA, USA).

Amplification reactions were conducted in duplicate using a QuantStudio™ 5 Real-Time PCR System (Applied Biosystems, Carlsbad, CA, USA), including non-template controls. Cycle threshold (Ct) values were automatically calculated using QuantStudio™ software, with threshold settings determined by default parameters.

Normalization of target miRNA expression levels was performed using miR-320 as an endogenous reference [22,23], chosen for its stable expression across samples. To ensure reliability, cases showing extreme Ct values of the reference miRNA (miR-320 < 16 or >25) were excluded from the analysis to minimize bias related to technical variability.

Relative expression was evaluated using ΔCt values (Ct target—Ct reference), with lower ΔCt indicating higher miRNA expression. These normalized ΔCt values were used for statistical comparisons and regression models.

### 2.3. Statistical Analysis

Associations between each miRNA and sepsis were estimated fitting linear models with ΔCt as outcome. The estimated coefficients and the corresponding 95% confidence intervals (95% CIs) were obtained. Analyses were conducted both unadjusted and adjusted for potential confounding factors, including age, sex, and time from death to autopsy.

All statistical tests were two-sided. To account for multiple testing, we computed Benjamini–Hochberg (BH) adjusted *p*-values. A miRNA was considered to be differentially expressed between sepsis and control deaths when the BH-adjusted *p*-value was ≤0.05. Statistical analyses were performed using R software, version 4.5.1.

## 3. Results

### 3.1. Characteristics of the Study Sample

Out of the initial 58 post-mortem peripheral whole-blood samples (32 sepsis-related deaths and 26 non-infective controls), 13 were excluded due to extreme Ct values for the reference miRNA (miR-320 Ct < 16 or Ct > 25), resulting in a final analytical cohort of 45 cases. The excluded cases comprised six sepsis-related deaths and seven controls. The final cohort therefore included 26 sepsis-related deaths and 19 non-infective controls.

As summarized in Table 1, the median age was 70 years (IQR: 65–83) in the sepsis group and 60 years (IQR: 52–77) in the control group (*p* = 0.118). The proportion of males was 54% in sepsis cases and 84% among controls (*p* = 0.054). The median post-mortem interval (PMI; days from death to autopsy) was 4 days (IQR: 2–7) in sepsis cases and 3 days (IQR: 3–4) in controls (*p* = 0.564). Overall, the study population was elderly, consistent with the demographics typically observed in medico-legal autopsy series.

### 3.2. miRNA Expression and Association Analyses

**Figure 1:**
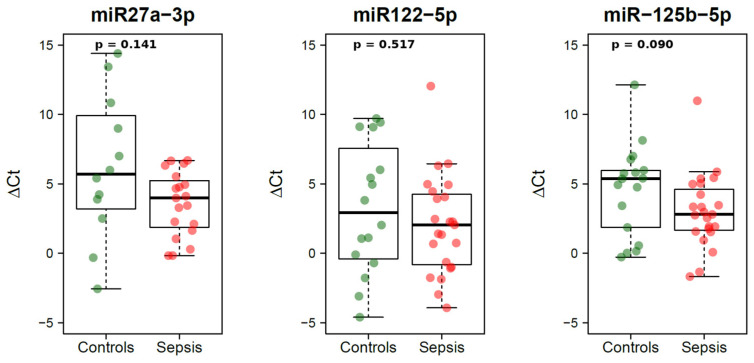
Boxplots of ΔCt values of miR-27a-3p, miR-122-5p, and miR-125b-5p in sepsis-related deaths (red) and non-infective controls (green). Lower ΔCt values indicate higher miRNA expression. Mann–Whitney *p*-values are displayed above each panel: miR-27a-3p *p* = 0.141; miR-122-5p *p* = 0.517; miR-125b-5p *p* = 0.090.

All three miRNAs (miR-27a-3p, miR-122-5p, and miR-125b-5p) showed lower median ΔCt values in the sepsis group compared with controls, indicating a trend toward higher expression in sepsis-related deaths (Table 1). Figure 1 shows the distribution of ΔCt values in the two groups, with the corresponding univariable Mann–Whitney *p*-values reported above each panel.

In linear regression analyses, only miR-125b-5p and miRNA-27a-3p showed evidence of association with sepsis-related death status (vs. controls) in the adjusted model (BH-adjusted *p* = 0.016 and 0.041, respectively) (Table 2).

## 4. Discussion

In this study, we evaluated whether selected blood-based microRNAs differ between sepsis-related deaths and non-infective controls in a post-mortem forensic cohort. Among the three miRNAs analyzed, only miR-125b-5p and miR-27a-3p showed a statistically significant association with sepsis-related deaths, suggesting a potential role in the molecular response to systemic infection. The other target, miR-122-5p, displayed a similar overall expression pattern, with higher levels in sepsis cases compared to controls, although these differences did not reach statistical significance. These findings should be interpreted as exploratory and hypothesis-generating, given the limited sample size and the lack of external validation.

Notably, miR-27a-3p showed an association with sepsis status in the adjusted model (BH-adjusted *p* = 0.041); however, the confidence interval remained relatively wide, indicating limited precision and reinforcing the need for replication in larger cohorts.

### 4.1. Comparison with Previous Studies

The observed overexpression of miR-125b-5p in sepsis-related deaths aligns with several reports describing its involvement in inflammatory pathways and immune modulation during sepsis.

Previous in vivo and in vitro studies have shown that miR-125b-5p regulates key mediators such as IL-1β, TNFα and NF-κB, acting as a modulator of the inflammatory response [14,15,16,24,25,26]. Moreover, increased circulating levels of this miRNA have been described in septic patients, often correlating with disease severity, inflammation and unfavorable outcomes [10,11,27].

Our findings extend this evidence to the post-mortem setting, suggesting that miR-125b-5p remains detectable and differentially expressed after death, thereby supporting its potential stability and applicability in forensic investigations.

In contrast, miR-122-5p did not show statistically supported differences in our cohort. For miR-27a-3p, the evidence was more tentative: while the association emerged in the adjusted regression model after controlling for age, sex, and post-mortem interval (BH-adjusted *p* = 0.041), the confidence interval was wide, indicating limited precision. This pattern suggests that any potential signal for miR-27a-3p should be interpreted cautiously and requires confirmation in larger cohorts.

Both miR-122-5p and miR-27a have been previously implicated in sepsis and systemic inflammation. In particular, miR-122-5p has been associated as a marker of hepatocellular injury [17,18]. Recently, it has been associated with renal injury, in a study conducted by Wang and colleagues on rats [28]. Furthermore, several studies revealed the involvement of miR-122 in sepsis pathogenesis, also suggesting a role as potential biomarker and predictor of severity and mortality [12,29,30].

On the other hand, miR-27a has been associated with the regulation of endothelial and immune responses, suggesting its involvement in the development of septic shock [19].

A predictive role in septic patients has also been demonstrated for miR-27a, based on its correlation with oxidative stress and mortality [13].

However, the precise role of miR27a in sepsis remains controversial across different models and studies. While some reports show miR-27a is upregulated and promotes inflammation and organic dysfunction, particularly in lung injury [31], other suggest a protective role, noting that its levels are reduced in septic mice and that miR-27a transfection alleviates acute lung injury induced by sepsis [20,32]. Such discrepancies may reflect differences in species, tissue type, timing of sample collection, and analytical/normalization strategies.

Importantly, for both miR-122-5p and miR-27a, the majority of current evidence derives from in vivo/in vitro studies or clinical cohorts rather than cadaveric samples; therefore, their relevance to post-mortem expression remains uncertain. Overall, our results provide the clearest support for miR-125b-5p, whereas findings for miR-27a-3p should be considered preliminary and in need of replication, and miR-122-5p did not show evidence of differential expression in this cohort. Additional studies in larger cohorts, ideally with independent validation and integration with complementary post-mortem markers, are required to conclusively determine post-mortem stability and diagnostic utility.

### 4.2. Forensic and Diagnostic Implications

From a forensic perspective, the identification of molecular markers that remain informative after death is of particular interest, especially in conditions such as sepsis, where morphological findings and the endogenous inflammation mediators are often nonspecific [23].

The accurate diagnosis of sepsis in forensic pathology is of paramount importance, particularly where nosocomial infections leading to sepsis frequently become subjects of litigation and medical malpractice investigations.

In order to fill this diagnostic gap, different methods have been researched to obtain biological markers that could serve as a reference for a more certain diagnosis of sepsis.

Since macroscopic and histological evidence, or even microbial isolation, often proves insufficient to formulate a specific diagnosis, various studies over the years have investigated potential and valid supplementary instruments. For example, several immunohistochemical markers, as well as cytokines involved in inflammatory processes and the host systemic response, have been tested as targets for the post-mortem diagnosis of sepsis [33]. Finally, of course, several panels of miRNAs were evaluated to identify stable and reliable markers in this challenging setting.

The use of blood-based miRNAs extracted from peripheral blood may offer practical advantages in forensic contexts: they are accessible, relatively stable, and can reflect systemic biological processes occurring before death.

The association observed for miR-125b-5p (and, to a lesser extent, miR-27a-3p) in adjusted exploratory models strengthens the potential utility of miRNA profiling as a complementary tool in post-mortem diagnostics.

If validated in larger cohorts, miR-125b-5p—or a panel including multiple miRNAs—could contribute to improving diagnostic confidence in suspected sepsis-related deaths, particularly in cases with limited or ambiguous histopathological evidence. The possibility of using this marker, even when adjusted for forensic confounders like PMI, supports the potential for developing a reliable molecular toolkit, paving the way for a more comprehensive diagnostic approach that integrates genomic and proteomic data.

### 4.3. Study Limitations and Future Perspectives

Several limitations of our work must be acknowledged. First, the sample size was relatively small, which may have limited statistical power to detect modest associations, particularly for miR-27a-3p and miR-122-5p.

Second, the study population was predominantly older (median age 60–70 years), in line with the epidemiology of sepsis and the typical forensic autopsy case-mix; because miRNA expression may vary with age, we adjusted all regression models for age, but residual confounding cannot be excluded.

Third, 13/58 cases were excluded based on predefined quality-control thresholds for the reference miRNA (miR-320), which was necessary to reduce technical variability in ΔCt-based normalization but may have introduced selection bias. In post-mortem whole-blood material, extreme reference Ct values may reflect pre-analytical or technical factors (e.g., variable RNA integrity/degradation, PCR inhibition, or hemolysis) rather than the underlying cause of death; however, residual selection bias cannot be excluded.

All samples were collected and stored following standardized post-mortem handling procedures to ensure miRNA integrity. However, despite the intrinsic stability of miRNAs, once post-mortem changes are initiated, the persistence and intensity of sepsis-related biological processes may variably affect the expression of individual miRNAs, making their post-mortem interpretation complex.

Future studies should therefore aim to validate these findings in larger, prospective cohorts, ideally combining molecular, histological, and immunohistochemical data. The integration of miRNA expression profiles with other emerging post-mortem biomarkers may further enhance the diagnostic accuracy for sepsis in the forensic setting.

In this regard, this work represents a preliminary, exploratory phase of a broader research project aimed at developing a multimodal diagnostic framework for the post-mortem identification of sepsis. The next phase will involve immunohistochemical investigations targeting key inflammatory mediators and sepsis-related proteins, in order to correlate molecular alterations with histopathological findings. The integration of molecular (miRNA-based) and protein-level (immunohistochemical) data may provide a more comprehensive understanding of the pathophysiological mechanisms underlying sepsis and strengthen the reliability of its diagnosis in the forensic setting.

## 5. Conclusions

Despite its exploratory nature, this study shows that circulating microRNAs can be reliably quantified in post-mortem peripheral blood samples and may display differential expression patterns between sepsis-related and non-infective deaths. In adjusted exploratory models controlling for age, sex, and post-mortem interval, and accounting for multiple testing (BH correction), miR-125b-5p showed the clearest and most consistent association with sepsis status, while miR-27a-3p provided more tentative evidence characterized by limited precision. miR-122-5p did not show statistically supported differences in this cohort.

Overall, miR-125b-5p appears to be a promising candidate for an objective, complementary biomarker in the forensic setting, where traditional morphological findings are often non-specific. These preliminary findings support the feasibility of miRNA-based approaches and encourage validation in larger cohorts. Expanding sample size and integrating molecular analyses with immunohistochemical and histopathological evaluations will be essential steps toward developing a standardized multimodal diagnostic framework for post-mortem sepsis identification.

## Figures and Tables

**Table 1:** Demographic and clinical characteristics of the study sample (final analytical cohort).

	Sepsis (N = 26)	Controls (N = 19)	*p*-Value *
Age, median (IQR)	70 (65, 83)	60 (52, 77)	0.118
Days since death, median (IQR)	4 (2, 7)	3 (3, 4)	0.564
Sex, males, N (%)	14 (54)	16 (84)	0.054
miRNA-27a-3p, ΔCt, median (IQR)	4.0 (1.9, 5.2)	5.7 (3.5, 9.5)	0.141
miRNA-122-5p, ΔCt, median (IQR)	2.0 (−0.8, 4.3)	2.9 (−0.3, 6.8)	0.517
miRNA-125b-5p, ΔCt, median (IQR)	2.8 (1.7, 4.6)	5.4 (1.9, 6.0)	0.090

* Mann–Whitney test for all variables except sex (Fisher’s exact test). IQR = interquartile range. Lower ΔCt values indicate higher miRNA expression.

**Table 2:** Associations between miRNAs expression and sepsis-related death from unadjusted and adjusted linear regression models. Coefficients represent difference in ΔCt mean in sepsis compared to control deaths.

	Unadjusted Model			Adjusted Model *		
	Coefficient (95% CI)	*p*-Value	BH-Adjusted *p*-Value	Coefficient (95% CI)	*p*-Value	BH-Adjusted *p*-Value
MiRNA-27a-3p	−3.6 (−7.4, 0.2)	0.061	0.091	−5.1 (−9.5, −0.6)	0.027	0.041
MiRNA-122-5p	−2.3 (−5.2, 0.6)	0.116	0.116	−2.3 (−5.9, 1.2)	0.191	0.191
MiRNA-125b-5p	−2.5 (−4.6, −0.5)	0.016	0.048	−3.4 (−5.7, −1.1)	0.005	0.016

* Model adjusted for age, sex, and days since death.

## Data Availability

The data presented in this study are available on request from the corresponding author. The data are not publicly available due to investigative secrecy.

## Abbreviations

The following abbreviations are used in this manuscript:

cDNA	Complementary deoxyribonucleic acid
Ct	Cycle threshold
EDTA	Ethylenediaminetetraacetic acid
IL-1β	Interleukin-1β
IQR	Interquartile Range
NF-κB	Nuclear Factor kappa-light-chain-enhancer of activated B cells
PMI	Post-mortem Interval
qRT-PCR	Quantitative Reverse Transcription Polymerase Chain Reaction
RNA	Ribonucleic acid
TNFα	Tumor necrosis factor α
WHO	World Health Organization
